# An Audit of Venous Thromboembolism (VTE) Risk Assessment in an NHS Trust Mental Health Inpatient Setting

**DOI:** 10.1192/bjo.2024.583

**Published:** 2024-08-01

**Authors:** Manjula Atmakur, Sururat Ibrahim, Oluwakemisola Adedipe

**Affiliations:** 1 Black Country Healthcare NHS Foundation Trust, Wolverhampton, United Kingdom; 2 Black Country Healthcare NHS Foundation Trust, Dudley, United Kingdom; 3 Royal Wolverhampton NHS Trust, Wolverhampton, United Kingdom

## Abstract

**Aims:**

To assess compliance with National Institute of Clinical Excellence (NICE) guidance (NG 89) recommendations on VTE risk assessment for mental health inpatients in Black Country Healthcare NHS Foundation Trust (BCHFT).

**Methods:**

NICE guidance (NG89) set out recommendations on VTE risk assessment for adult psychiatric inpatients. It recommends that:

- All adult psychiatric inpatients have their VTE risk assessed as soon as possible after admission or at the first consultant's review.
- All patients have their VTE risk re-assessed at the point of consultant's review or if their clinical condition changes.
- Any patient found to be at VTE risk should be considered for prophylaxis with Low Molecular Weight Heparin III (LMWH III), if the thrombotic risk outweighs the bleeding risk. Fondaparinux sodium should be considered in those with contraindications to LMWH III.
- VTE pharmaco-prophylaxis should be continued until the patient is no longer at increased VTE risk.

Using these recommendations as standards, we retrospectively evaluated inpatient clerking charts and progress notes of 49 inpatients across all the 23 wards in the Trust. Data were collected using a standardised audit tool on concordance with these standards to check how many patients had VTE risk assessment within 24 hours of their admission, whether patients had VTE risk re-assessment at any point during admission, whether the patients found to have increased VTE risk at admission were commenced on pharmaco-prophylaxis. Data was also collected to see if the patients commenced on VTE pharmaco-prophylaxis were re-assessed for continued need of the prophylaxis.

**Results:**

30 patients (59.2%) were risk assessed for VTE within 24 hours of admission. Only 2% of patients had VTE re-assessment while on admission, but there was no record of the indication for this. All the 3 patients (6.1%), found to be at VTE risk on admission, were prescribed pharmaco-prophylaxis, but none of them had a VTE re-assessment to determine the prophylaxis’ continued need.

**Conclusion:**

The trust’s compliance with NICE recommendation for VTE risk assessment is below standard. We felt the trust's compliance is a reflection of the medical staff's awareness on the importance of VTE risk assessment in mental health settings, and also lack of Trust's policy on VTE risk assessment. Recommendations were suggested to include VTE risk assessment in the junior doctors’ induction programme and for the trust to have a VTE policy that factors in mental health risk factors.

